# Bisindolylpyrrole triggers transient mitochondrial permeability transitions to cause apoptosis in a VDAC1/2 and cyclophilin D-dependent manner via the ANT-associated pore

**DOI:** 10.1038/s41598-020-73667-z

**Published:** 2020-10-12

**Authors:** Masami Koushi, Yasunori Aoyama, Yoshiko Kamei, Rei Asakai

**Affiliations:** Department of Morphophysiology, Faculty of Pharmaceutical Sciences, Josai International University, 1 Gumyo, Togane, Chiba 283-8555 Japan

**Keywords:** Apoptosis, Kinases

## Abstract

Bisindolylpyrrole at 0.1 μM is cytoprotective in 2% FBS that is counteracted by cyclosporin-A (CsA), an inhibitor of cyclophilin-D (CypD). We hypothesized that the cytoprotective effect might be due to transient mitochondrial permeability transition (tPT). This study tested the hypothesis that bisindolylpyrrole can trigger tPT extensively, thereby leading to cell death under certain conditions. Indeed, CsA-sensitive tPT-mediated apoptosis could be induced by bisindolylpyrrole at > 5 μM in HeLa cells cultured in 0.1% FBS, depending on CypD and VDAC1/2, as shown by siRNA knockdown experiments. Rat liver mitochondria also underwent swelling in response to bisindolylpyrrole, which proceeded at a slower rate than Ca^2+^-induced swelling, and which was blocked by the VDAC inhibitor tubulin and the ANT inhibitor bongkrekate, indicating the involvement of the ANT-associated, smaller pore. We examined why 0.1% FBS is a prerequisite for apoptosis and found that apoptosis is blocked by PKC activation, which is counteracted by the overexpressed defective PKCε. In mitochondrial suspensions, bisindolylpyrrole triggered CsA-sensitive swelling, which was suppressed selectively by pretreatment with PKCε, but not in the co-presence of tubulin. These data suggest that upon PKC inactivation the cytoprotective compound bisindolylpyrrole can induce prolonged tPT causing apoptosis in a CypD-dependent manner through the VDAC1/2-regulated ANT-associated pore.

## Introduction

The mitochondrial permeability transition (PT) occurs in response to high matrix Ca^2+^ and reactive oxygen species. When the PT is prolonged, it allows diffusion of key respiratory metabolites across the inner mitochondrial membrane, resulting in energetic failure and cell death^1−3^. The PT also occurs transiently^4−7^, which was suggested to function as a Ca^2+^ releasing channel for Ca^2+^-overloaded mitochondria^4,8,9^. Cyclophilin-D (CypD) regulates the PT pore (PTP) and is involved in cell death in ischemia–reperfusion injury and neurodegenerative diseases, as proved by the inhibition of mitochondrial swelling and cell death with cyclosporin-A (CsA)^10,11^ and later with CypD ablation^12−15^. However, the PT also occurs Ca^2+^-independently and CsA/CypD-insensitively. This phenomenon was hypothesized to take place when misfolded mitochondrial membrane proteins, responsible for pore formation, exceed CypD^16^. A classic model for the PTP is a complex of the voltage-dependent anion channel (VDAC) in the outer mitochondrial membrane, and the adenine nucleotide translocase (ANT) in the inner mitochondrial membrane^17^, although their essential role in the PTP has been challenged by studies in which they were genetically ablated^18,19^. F-ATP synthase has recently attracted a great deal of attention as a candidate structural component of the pore, where it can be transformed into a Ca^2+^-dependent large conductance channel^20−24^, although controversy exists regarding the molecular identity of the PTP^25,26^.

A large body of evidence shows that VDAC proteins have a pro-apoptotic activity or pro-survival activity, depending on their binding partners, which influence the VDAC conductance^27^. Although it was documented that VDAC1, the principal isoform, involves PTP-mediated apoptosis, occurring upon endostatin^28^ and selenite treatment^29^, how VDAC regulates the PTP remains to be elucidated.

Protein kinase C (PKC), especially the ε isoform (PKCε), is known to mediate preconditioning and protect hearts or cardiocytes from ischemic injury^30−34^. One of the PKCε targets may be VDAC1, since this isoform exists as a complex with VDAC1 and can prevent Ca^2+^-induced mitochondrial swelling through VDAC1 phosphorylation^33^.

On the other hand, ANT regulates the sensitivity of the pore to calcium approximately threefold as revealed in the genetic elimination of ANT^18^. ANT ligands, atractyroside and bongkrekate, are well known to stabilize the translocase in opposite conformations to induce or prevent mitochondrial swelling, respectively^35,36^. In addition, ANT1 was reported to confer voltage-sensitivity to the PTP^37^. Recently, patch-clamp studies with ATP synthase c-subunit knockout mitochondria demonstrated channels sensitive to CsA and bongkrekate, although they were found to have significantly lower conductance in comparison to classic PTP^38^. In addition, it was demonstrated that mouse cells lacking all three *Ant* genes and their mitoplasts completely lack PTP activity^39^. These reports strongly suggest that ANT may form an alternative form of the PTP.

In the preliminary studies to elucidate the cytoprotective mechanism of bisindolylmaleimide I (BMI) and bisindolylmaleimide V^40^ and its derivative 3, 4-Bis(5-methyl-1H-indol-3-yl)-*N*-methylpyrrole (BP)^41^, we found that the cytoprotective effect against Ca^2+^-mediated cell death can be, paradoxically, counteracted by CsA, a PTP closer. We hypothesized that the cytoprotective mechanism is due to a transient PT (tPT) triggered by these compounds, which allows for mitochondrial calcium release and for prevention of the mitochondria from swelling. If this is true, under certain conditions, such tPT could be prolonged and ultimately converted to permanent opening due to the loss of essential matrix molecules such as NAD(H) and cell death. This hypothesis was explored by treating with BP at > 5 μM in HeLa cells cultured under 0.1% FBS, transfected with the green fluorescent protein (GFP)-cytochrome *c* gene^42^. We show that BP induces prolonged tPT, leading to apoptosis, through VDAC in association with ANT upon the absence of PKC (especially PKCε) activity due to low FBS.

## Results

### BP causes apoptosis in a CsA/CypD-dependent manner through tPT under low FBS conditions

**Figure 1:**
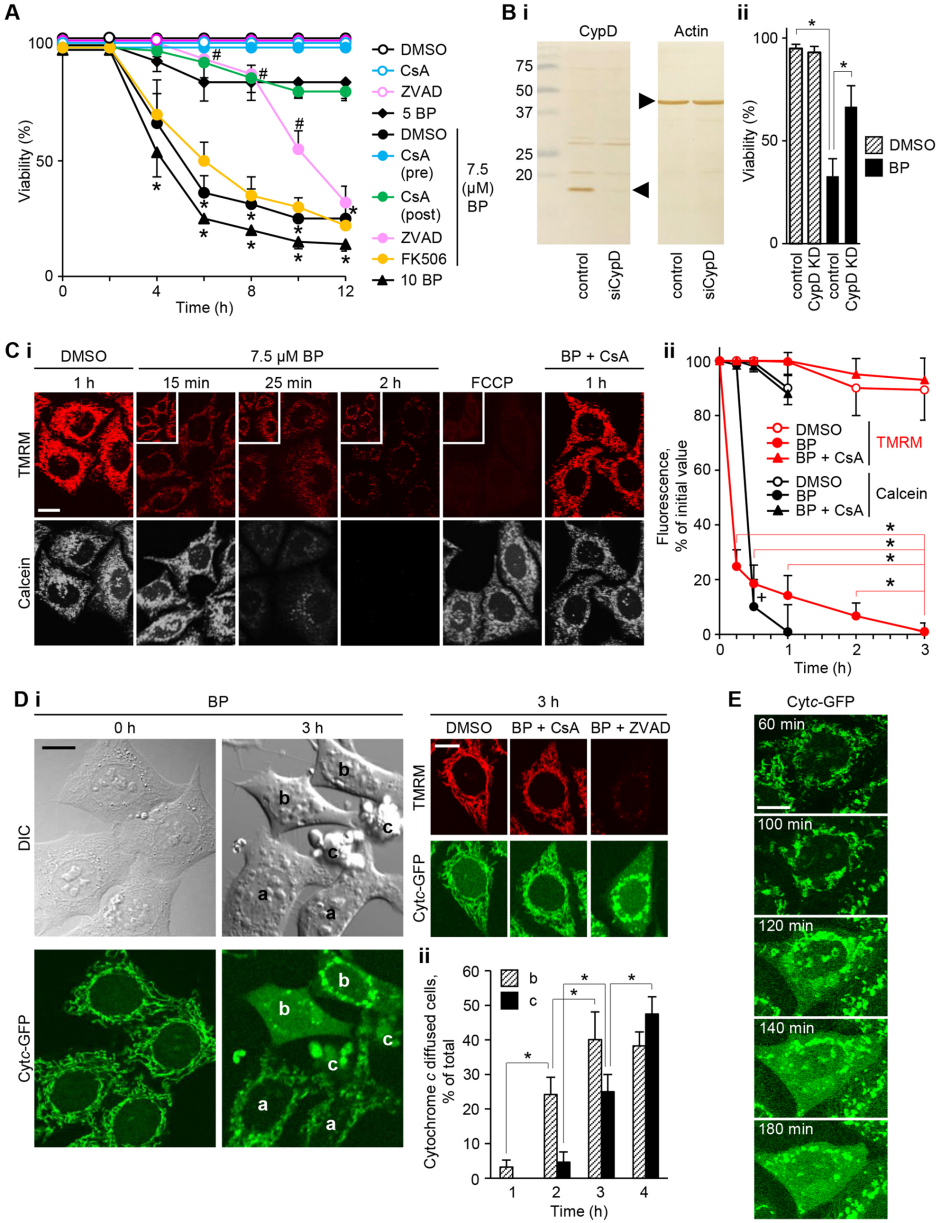
Bisindolylpyrrole (BP) induces tPT, before apoptosis, under 0.1% FBS in HeLa cells transfected with the GFP-cytochorome *c* gene. (**A**) Cells were incubated for 12 h in the presence or absence of BP from 5 to 10 μM with or without 3 μM CsA, which was added 30 min before BP addition (pre) or at least 1 h later (post), 20 μM ZVAD or 10 μM FK506. Data are representative of three separate experiments (error bars represent the mean ± SD; *p < 0.005 relative to control and to CsA plus BP; #*p* < 0.005 relative to 7.5 μM BP at the same time points). (**B**) Effect of CypD knockdown on apoptosis. (i) Immunoblotting with anti-CypD and actin antibodies of lysates from cells transfected with siRNA targeting CypD (siCypD) or control siRNA. (ii) Cell viability was determined 8 h after treatment with 7.5 μM BP. *p < 0.005. (**C**) The effects of 7.5 μM BP with or without CsA on mitochondrial entrapped calcein (signals pseudo colored in white) and TMRM signals (red; enhanced images in the insets); the membrane potential upon treatment with 0.5 μM FCCP was considered as a level of mitochondrial depolarization (i). Quantification of calcein and TMRM signals over 3 h (initial fluorescence intensities were normalized for comparative purposes; *p < 0.005 compared with BP treatment at 3 h; + *p* < 0.001 compared with control at the same time point by Welch’s *t*-test with Bonferroni’s correction; *n* = 100 cells) (ii). (**D**) Signals for mitochondrial cytochrome *c*-GFP (Cyt*c*-GFP) at 3 h; different apoptotic stages of the cells are present, with (*a*) mitochondrial clustering around the nuclei, (*b*) cytochrome *c*-GFP release into the cytoplasm, and (*c*) apoptotic blebs (differential interference contrast micrograph [DIC]). Representative fields were randomly imaged (i). Quantification of cells with cytochorome *c*-GFP release (*b*) and apoptotic bodies (*c*) over 4 h (*p < 0.001 by Welch’s *t*-test with Bonferroni’s correction; *n* = 100 cells) (ii). (**E**) Time-lapse analysis of the release of cytochrome *c*-GFP from mitochondria to the cytoplasm and nucleus in response to BP treatment. Scale bars: 10 μm. Images were digitized using FV10-ASW software version 4.2a (https://www.olympus-lifescience.com).

To verify the hypothesis that BP can induce the PT that ultimately causes cell death, we searched for the experimental conditions using HeLa cells stably expressing GFP-tagged cytochrome *c*. The results are shown in Fig. 1A. BP at 5–10 μM induced cell death after 4–8 h in 0.1% FBS, in contrast with less than 5% of cell death in control cells. The type of cell death was morphologically apoptotic, and this was further supported by a significant delay in the onset of cell death by treatment with Z-VAD-fmk (ZVAD), a caspase inhibitor. When CsA was added either 30 min before the addition of BP or at least 1 h later, it blocked apoptosis, whereas the specific calcineurin inhibitor FK506 did not, suggesting PTP involvement.

To determine the involvement of CypD, we performed knockdown experiments with CypD-targeting small interfering RNA (siRNA). As shown in Fig. 1B, transfection with this siRNA effectively reduced the protein expression and this conferred remarkable resistance to BP-induced apoptosis, whereas treatment with the control siRNA had no effect. These findings justify the conclusion that the presence of CypD is a prerequisite for BP-induced, CsA-sensitive apoptosis.

Cells were loaded with calcein to directly observe the opening of the PTP with large conductance, and the cytoplsmic calcein signals were quenched with Co^2+^. Calcein signals were much stronger than cytochrome *c*-GFP signals and were not significantly affected by the latter signal. Calcein signals were lost at 25 min after treatment with 7.5 μM BP; in contrast, tetramethylrhodamine methyl ester (TMRM) signals decreased as early as 15 min after treatment and remained low over 2–3 h while still being significantly higher than those values in the cells treated with carbonyl cyanide-p-(trifluoromethoxy)phenylhydrazone (FCCP). These changes by BP were blocked by CsA pretreatment (Fig. 1C), indicating the induction of tPT.

We evaluated the cytochrome *c* release from mitochondria leading to apoptosis and observed, different apoptotic stages of the cells were evident at 3 and 4 h, with (*a*) mitochondrial clustering around the nuclei, (*b*) cytochrome *c*-GFP release into the cytoplasm and nucleus, and (*c*) apoptotic blebs; these changes were blocked by CsA (Fig. 1Di). A quantification analysis showed that the apoptosis was preceded by the release of cytochrome *c*-GFP, which occurrred after 2–3 h of BP treatment (Fig. 1Dii). A time lapse analysis showed that cytochrome *c*-GFP was released in the cytoplasm from the mitochondria approximately 2 h after BP treatment, which was almost simultaneously diffused into the nucleus (Fig. 1E). Keeping with the pivotal role of nuclear translocation of cytochrome *c* for caspase-independent nuclear apoptosis^43^, this nuclear accumulation of cytochrome *c*-GFP may account for the observation that the inhibition of apoptosis by ZVAD was observed for only about 8 h and virtually all of the cells underwent cell death until 11 h (Fig. 1A). These data suggest that BP treatment may induce prolonged tPT, which transitions to the persistent pore opening associated with cytochrome *c*-mediated apoptosis.

### Mitochondrial autophagy follows tPT and influences the progress of apoptosis

**Figure 2:**
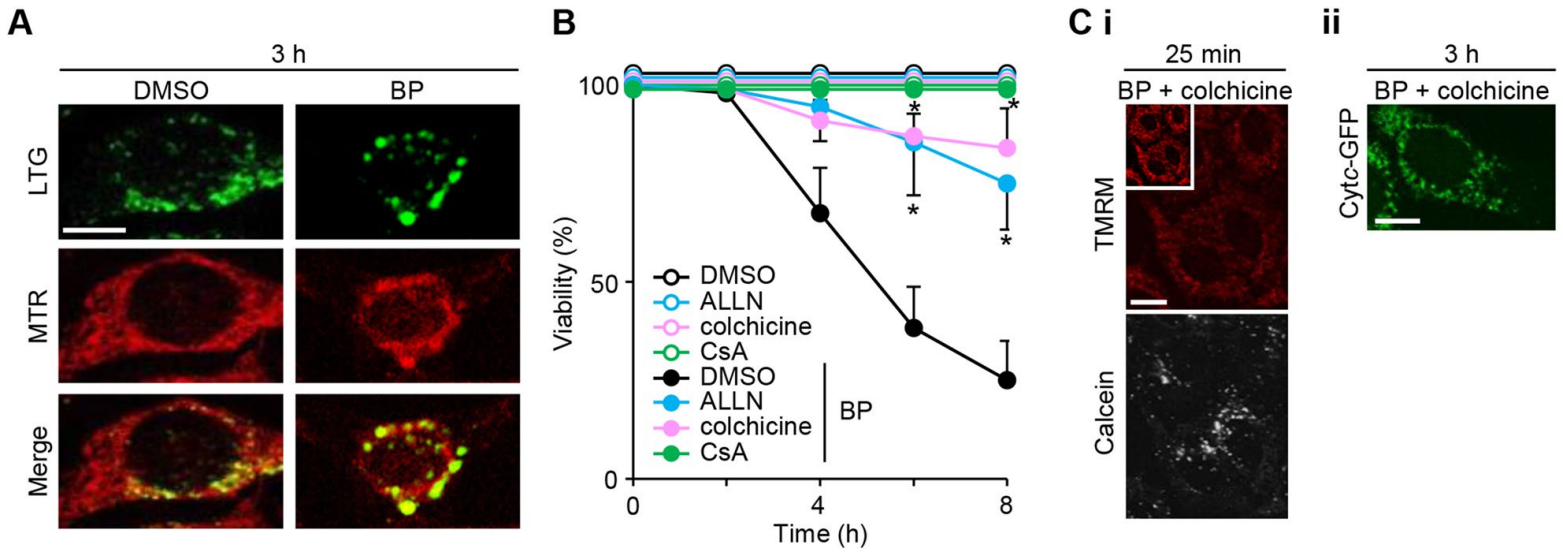
Involvement of mitochondrial autophagy in the apoptosis of BP. (**A**) Confocal images of lysosomes labeled with LysoTracker Green (LTG) and mitochondria labeled with MitoTracker Red (MTR), respectively, 3 h after treatment of DMSO or 7.5 μM BP. (**B**) Cell viability over 8 h in the presence of DMSO, 7.5 μM BP, 0.3 μg/ml colchicine, 50 μM ALLN, BP plus colchicine, or BP plus ALLN. Data are representative of three separate experiments. BP-induced cell death was delayed significantly by colchicine and ALLN (error bars represent the mean ± SD; *p < 0.01 compared with BP treatment at the same time points). (**C**) Confocal images for TMRM and calcein 25 min after BP treatment (i) and cytochrome *c*-GFP (Cyt*c*-GFP) 3 h after BP treatment (ii) in the presence of colchicine. Representative cells were randomly imaged. Scale bars: 10 μm. Images were digitized using FV10-ASW software version 4.2a (https://www.olympus-lifescience.com).

To determine whether the mitochondrial aggregation in BP-treated cells reflects the selective removal of impaired mitochondria by autophagosomes, or mitophagy, lysosomes and mitochondria were labeled with LysoTracker green (whose signals were much stronger than cytochrome *c*-GFP signals and were not significantly affected by the latter signal), and MitoTracker red, respectively. While control cells had small lysosomal signals with distribution distinct from the mitochondrial signals, BP-treated cells exhibited larger lysosomes in the perinuclear regions after 3 h, which were largely co-localized with mitochondrial signals (Fig. 2A), indicating autophagolysosomes containing mitochondria. When CsA was added 30 min before BP treatment, it blocked these mitochondrial changes (Fig. 1Ci), indicating that mitophagy follows PTP opening. We inquired about whether or not autophagy plays a role in the course of apoptosis and utilized colchicine (a microtubule polymerization inhibitor) and N-acetyl-Leu-Leu-Norleu-al (ALLN) (a peptide aldehyde inhibitor of lysosomal proteases, including cathepsin-B/L and calpains)^44^. Both reagents delayed significantly apoptosis (Fig. 2B), although all cells eventually died within 12 h. We then examined whether or not colchicine influences the BP-induced release of TMRM, calcein and cytochrome *c*. Colchicine did not affect the TMRM release and calcein release by BP (Fig. 2Ci), but it delayed the formation of autophagolysosomes and the release of cytochrome *c* in most cells (Fig. 2Cii), indicating that PTP opening precedes mitophagy and cytochrome *c* release. These data suggest that mitophagy may play a role in the progression of BP-induced apoptosis.

### Involvement of VDAC isoforms in BP-induced apoptosis

**Figure 3:**
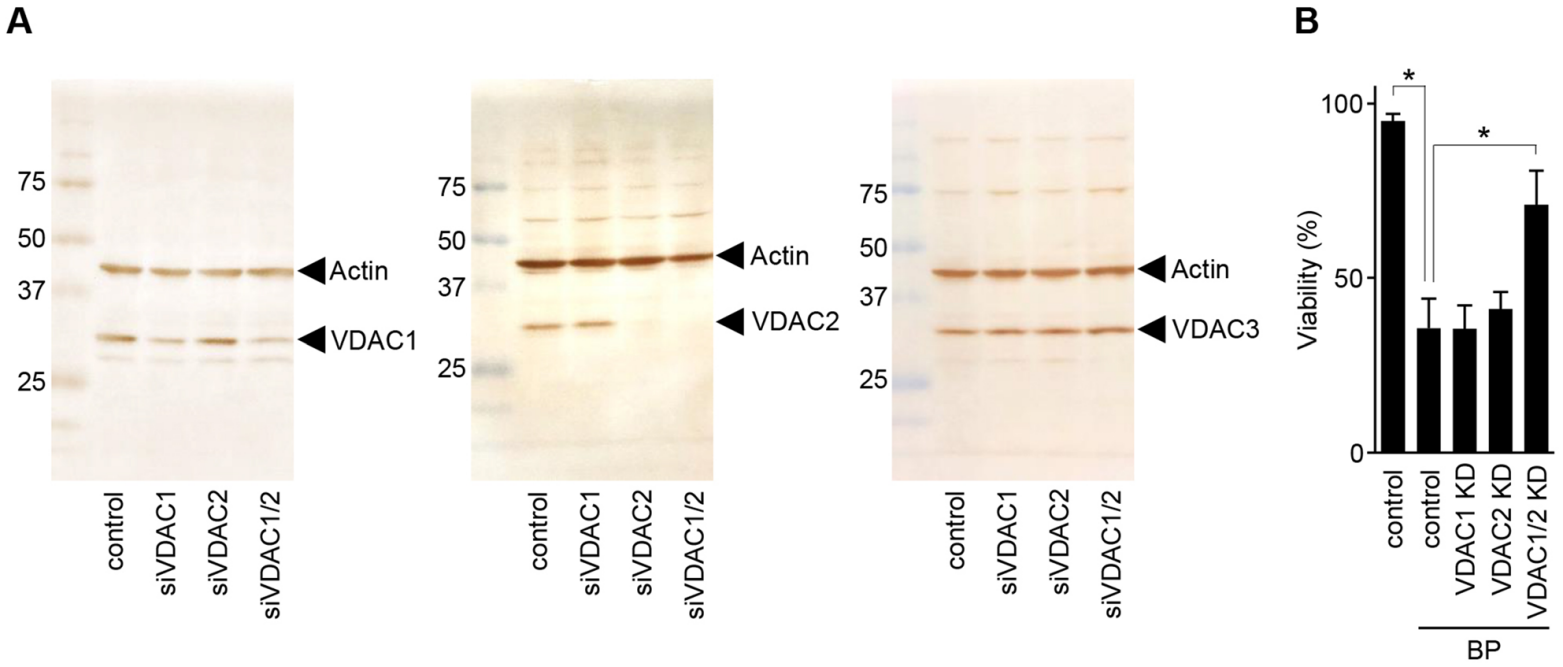
Effect of isoform-specific siRNA knockdown of VDAC1/2 on BP-induced apoptosis. (**A**) HeLa cells were transfected with siRNAs (non-target siRNA, VDAC1-specific siRNA, VDAC2-specific siRNA or VDAC1-specific siRNA plus VDAC2-specific siRNA). After 36 h, the protein expression of the two isoforms was assessed by immunoblotting with anti-VDAC1 (left panel), VDAC2 (middle panel), VDAC3 (right panel) and actin antibodies. Note the decrease in the specific VDAC isoforms after knockdown of each VDAC isoform. (**B**) At 36 h after transfection, HeLa cells were treated for 8 h with 10 μM BP or DMSO. Note that only double knockdown conferred remarkable resistance to apoptosis.

VDAC is a regulator of the PTP. To investigate whether or not BP-induced apoptosis is mediated by VDAC, we knocked down VDAC1 and/or VDAC2 using their specific siRNAs (siVDAC1 and siVDAC2). As shown in Fig. 3A, immunoblotting showed that transfection with each isoform of siVDAC1 and siVDAC2 effectively reduced the protein expression of the target VDAC isoforms without significantly affecting the non-target VDAC isoforms, including VDAC3. We observed that cells doubly transfected with siVDAC1 and siVDAC2 were remarkably resistant to BP-induced apoptosis (Fig. 3B), whereas those treated with siVDAC1 or siVDAC2 had no effect. These findings indicate that both isoforms of VDAC1 and VDAC2 can mediate the BP-induced, CsA-sensitive apoptosis.

### Properties of the BP-induced PTP opening in isolated rat liver mitochondria

**Figure 4:**
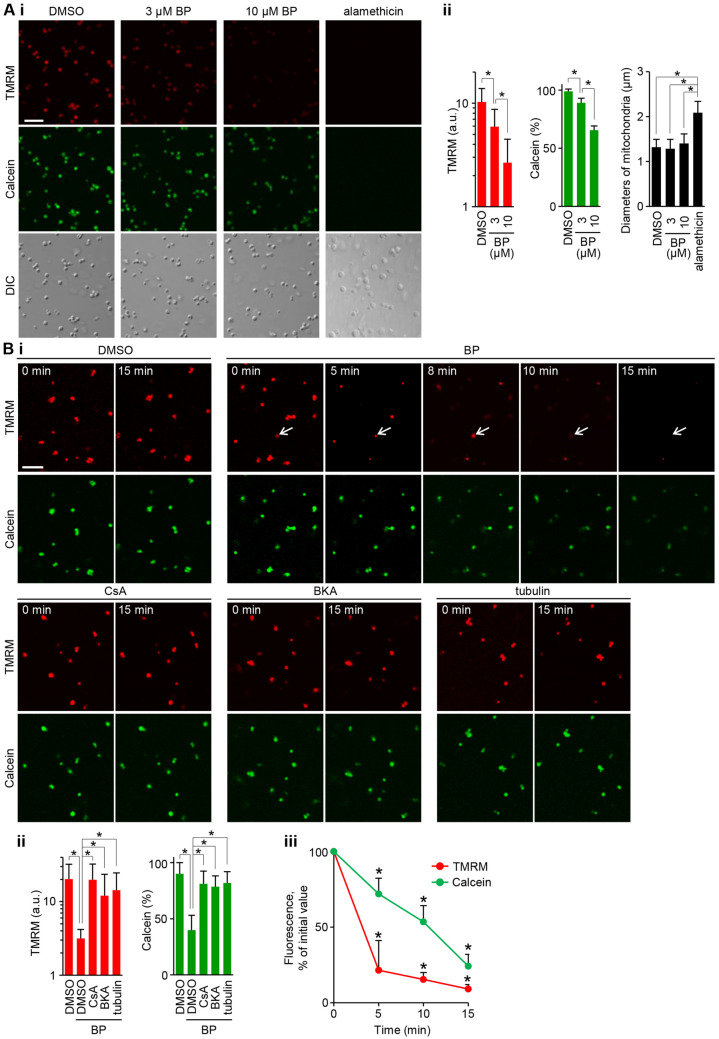
Properties of BP-induced PTP opening in isolated rat liver mitochondria energized with succinate, preloaded by TMRM and calcein. (**A**) The dose dependent effect of BP (3 and 10 μM) in mitochondrial suspensions. Mitochondria were also treated by 10 μg/ml alamethicin. (i) Representative confocal microscopic fields were randomly imaged at 10 min for TMRM and calcein signals and also the same fields were imaged by a differential interference contrast microscope (DIC). (ii) Quantification of fluorescence signals for TMRM (logarithmic scale) and calcein (linear scale) and of the mitochondrial diameters measured from DIC micrograph images (error bars represent the mean ± SD; *p < 0.01; *n* = 300 mitochondria). Note that BP-induced PTP opening was not accompanied by mitochondrial swelling. (**B**) Inhibitory effects of 3 μM CsA, 50 μM bongkrekate (BKA) and 50 nM tubulin on BP (10 μM)-induced pore opening in agar-embedded mitochondria. (i) Representative images of TMRM and calcein signals of mitochondria in the same areas at 0 and 15 min, and time-lapse images at 0, 5, 8, 10 and 15 min after BP treatment. Note TMRM signal fluctuations in the same mitochondrion (arrows), indicative of tPT. (ii) Quantification of images for fluorescence signals of TMRM (logarithmic scale) and calcein (linear scale) at 15 min (error bars represent the mean ± SD; *p < 0.01; *n* = 100 mitochondria). (iii) Quantification of time-lapse images (initial fluorescence intensities were normalized for comparative purposes; error bars represent the mean ± S.D; *n* = 100 mitochondria; *p < 0.005 in comparison to BP treatment at 0 min). Scale bars: 5 μm. Images were digitized using FV10-ASW software version 4.2a (https://www.olympus-lifescience.com).

To determine whether or not BP directly acts on mitochondria, experiments were performed with isolated rat liver mitochondria preloaded with TMRM and calcein. BP was able to trigger PTP opening dose-dependently at 3 and 10 µM in mitochondrial suspensions (Fig. 4A). The quantification of mitochondrial diameters based on differential interference contrast micrographs revealed no significant swelling during BP-induced PTP opening, in contrast to the striking swelling observed when mitochondria were treated with the nonspecific permeabilizing agent alamethicin (Fig. 4A).

Time course experiments were then performed over 20 min with mitochondria that were immobilized in agar^45^ As shown in Fig. 4Bi and iii, upon BP treatment, the TMRM signals started to drop within 2–5 min, and calcein release occurred over 15 min; both effects were completely blocked by 3 μM CsA (Fig. 4B). This slow calcein release was in contrast with the rapid release of calcein when mitochondrial swelling was induced by Ca^2+^ loading (Supplementary Fig. S1). These data suggest that the pore size induced by BP may be smaller than that of Ca^2+^.

To determine the involvement of VDAC in BP-induced pore opening, tubulin was used as a VDAC inhibitor^46^. BP was not able to trigger pore opening in the presence of 50 nM tubulin, as indicated by the inhibition of depolarization and calcein release (Fig. 4B), indicating VDAC involvement. Next, we assessed the involvement of ANT with bongkrekate, a membrane-permeant ANT ligand that promotes *m*-state conformation and inhibits pore opening^35^. Notably, bongkrekate prevented the release of TMRM and calcein signals by BP (Fig. 4B), indicating the involvement of ANT.

### PKC determines the susceptibility to BP-induced apoptosis

**Figure 5:**
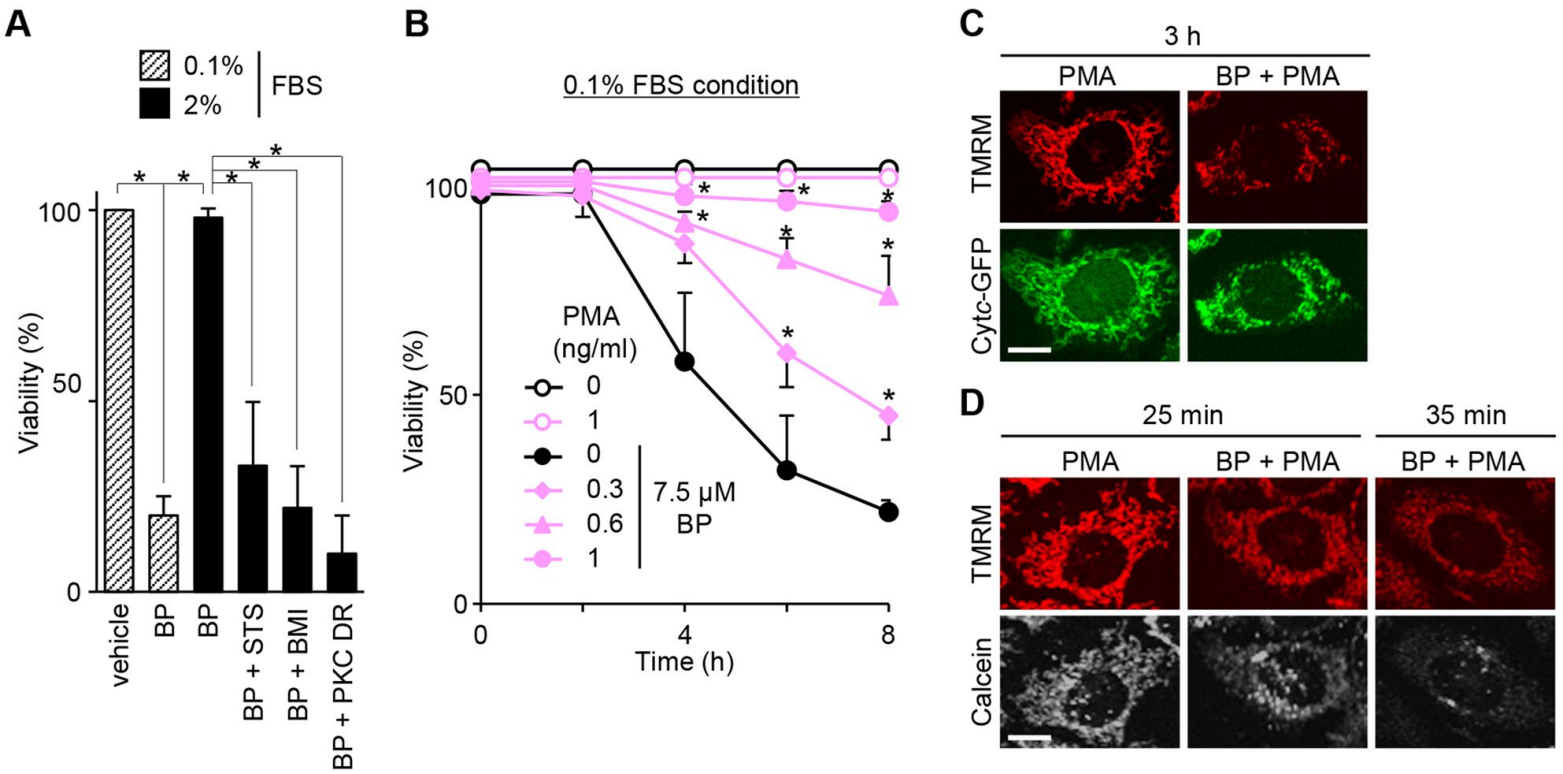
Involvement of PKC signaling in BP (7.5 μM)-induced apoptosis. (**A**) Effect of DMSO, 30 nM staurosporine (STS), 10 μM BMI or PKC downregulation (PKC DR) on the cytoprotective effect of 2% FBS against BP. Data are representative of three separate experiments (error bars represent the mean ± SD; *p < 0.005). (**B**) Dose-dependent apoptosis inhibition by PMA (0.3 to 1 ng/ml); data are representative of three separate experiments (error bars represent the mean ± SD; *p < 0.005 compared with BP treatment at the same time points). (**C**) Confocal images for TMRM and cytochrome *c*-GFP (Cyt*c*-GFP) at 3 h in cells treated with PMA or BP plus PMA. (**D**) Confocal images for TMRM and calcein at 25 and 35 min in cells treated with PMA or BP plus PMA. Representative fields were randomly imaged. Scale bars: 10 μm.

Cells under high FBS conditions (> 2%) were resistant to the induction of apoptosis by BP. What signaling pathways activated by FBS are involved in the resistance to BP? Because PKC is involved in the PTP-mediated pathological process^30−34^ we investigated whether PKC could modulate apoptosis induced by BP. In the presence of either staurosporine or BMI, PKC inhibitors, the cells became susceptible to BP even in 2% FBS (Fig. 5A). A second approach was then adopted, namely long-term treatment with 1 ng/ml phorbol 12-myristate 13-acetate (PMA) for 24 h, an established method that downregulates all PKC isozymes except ζ and λ, due to proteolytic degradation. The PKC-downregulated cells also became susceptible to BP (Fig. 5A). These data suggest that the cytoprotection of 2% FBS against BP may depend on the activation of PKC. If this is true, conversely, BP-induced apoptosis in 0.1% FBS will be reversed by the activation of PKC. In fact, PMA treatment reversed the sensitivity to apoptosis in a dose-dependent way, ranging from 0.3 to 1 ng/ml (Fig. 5B). These findings suggest that PKC signaling plays an essential role in the susceptibility to BP.

The possible effect of PKC activation by PMA was validated by confocal microscopic images for cytochrome *c*-GFP, TMRM and calcein. At 3 h after BP treatment, PMA inhibited the diffusion of cytochrome *c*-GFP signals in most of the cells and also apparently inhibited the formation of autophagolysosomes (Fig. 5C). However, PMA didn’t block the occurrence of tPT (Fig. 5D). Taking a close look, PMA was found to reduce the declining rates of both TMRM and calcein signals in comparison to those observed after treatment with BP alone (compare Fig. 5D with Fig. 1C at 25 min after BP treatment). One plausible mechanism underlying the PMA suppression of apoptosis is that PKC activation causes the reduction in the frequency or open period of tPT and this prevents presumably the release of NAD(H) from the matrix and consequently avoids the permanent PTP opening and cytochrome *c* release.

### PKCε involvement via VDAC

**Figure 6:**
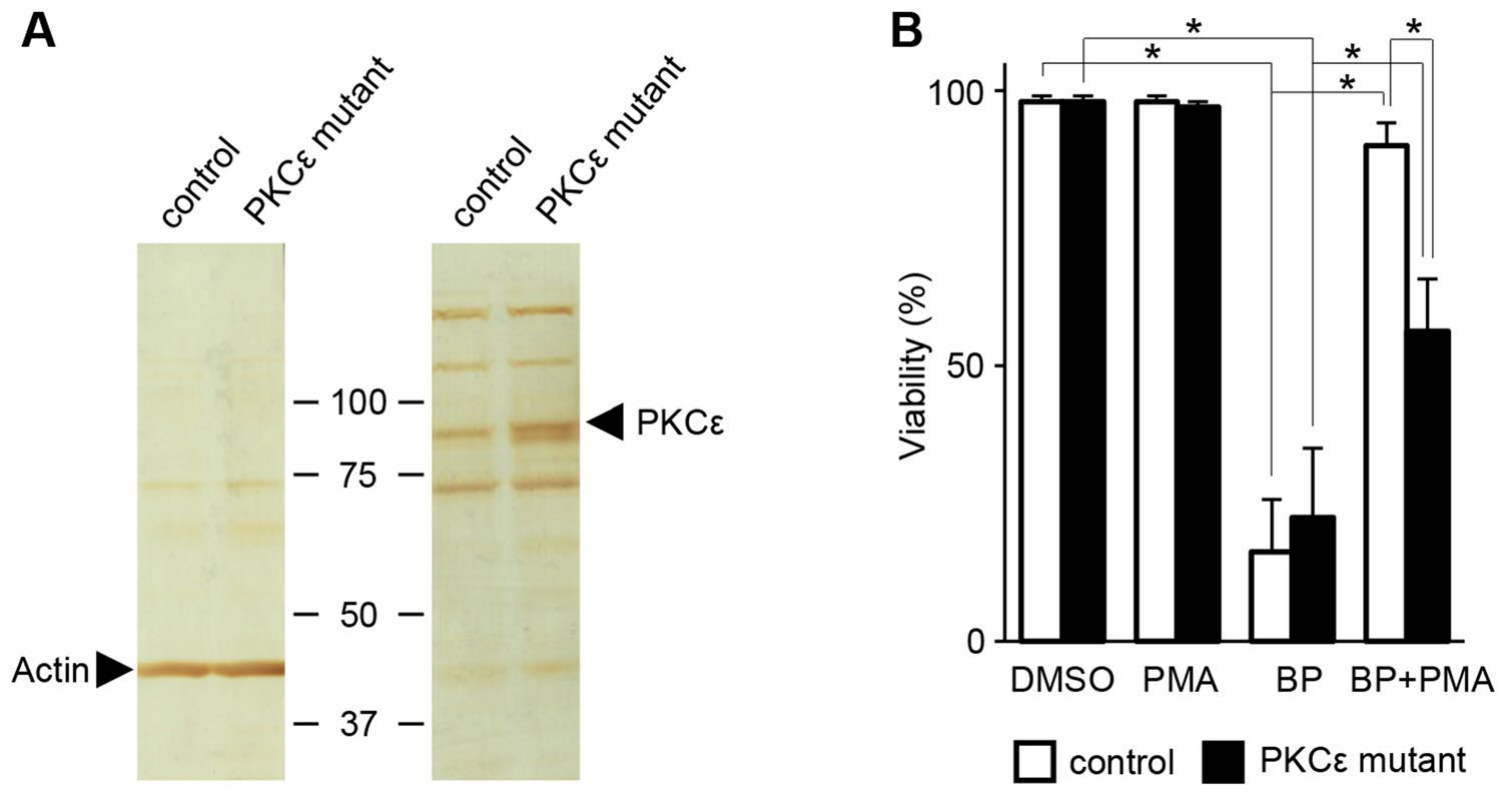
Inactive PKCε counteracts PMA-induced inhibition of the apoptosis by BP in HeLa cells. (**A**) Immunoblotting with anti-PKCε and actin antibodies of lysates from cells transfected with adenovirus expressing a kinase-inactive mutant of PKCε or vector control. (**B**) The inactive PKCε overexpressing cells and control cells were treated for 8 h with DMSO, 1 ng/ml PMA, 10 μM BP, or BP plus PMA, and cell viability was determined. Data are representative of three separate experiments (error bars represent the mean ± SD; *p < 0.005).

We focused on the PKC isozyme, PKCε, and validated its role in PMA-induced cytoprotection against BP using the HeLa cells overexpressing a kinase-deficient mutant of PKCε^47^ and control cells simply overexpressing the vector. Immunoblot analysis with PKCε antibody of cell lysates shows expression of an 84 kDa band (Fig. 6A), corresponding to mutant PKCε. In response to BP, both types of cells equally underwent apoptosis; however, upon treatment with 1 ng/ml PMA for PKC activation, cell viability in the inactive PKCε overexpressing cells was reduced to approximately half (*p* < 0.005) in comparison to control cells (Fig. 6B). This indicates a primary role of the PKCε signaling pathway.

**Figure 7:**
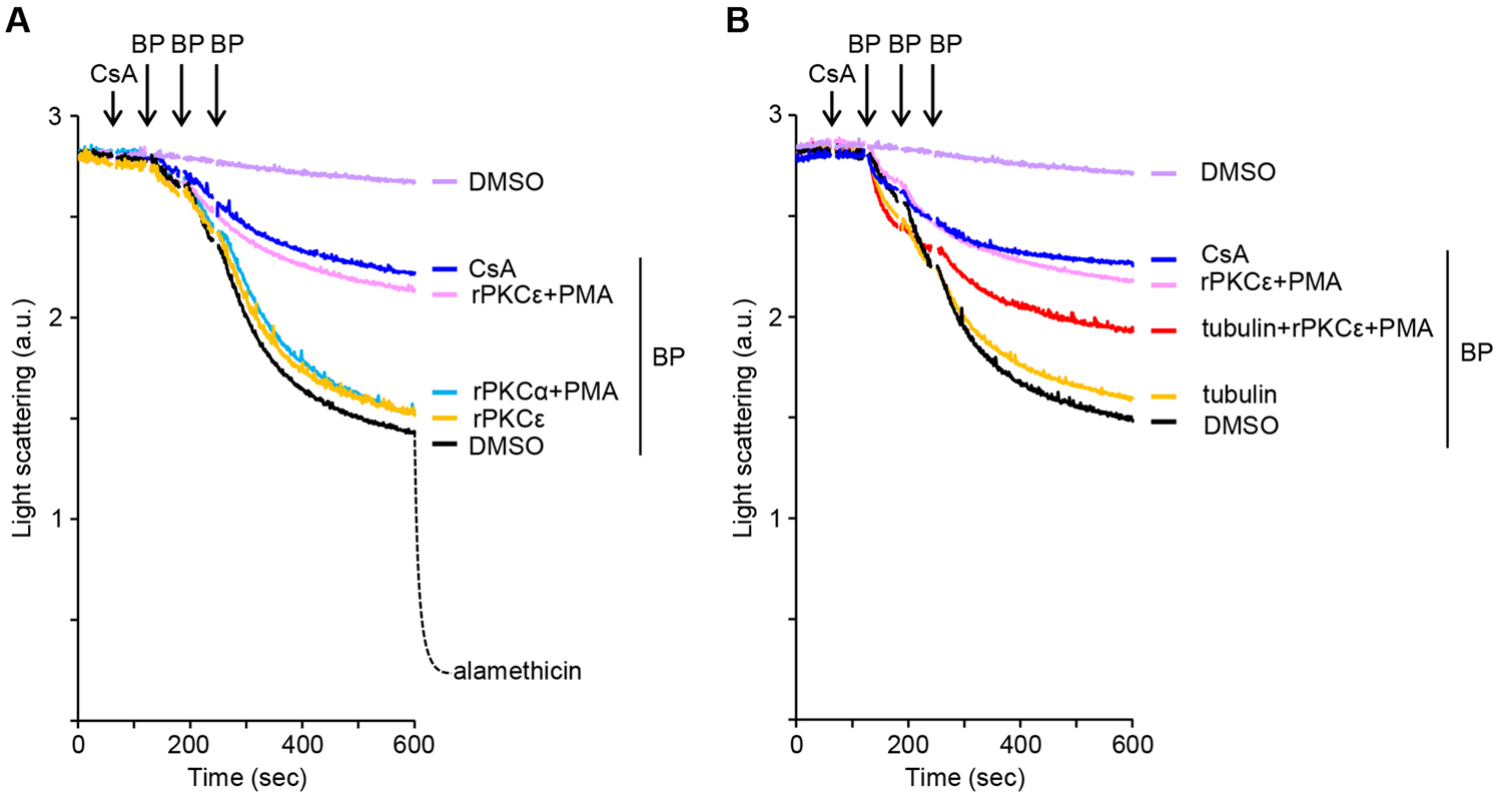
BP induces mitochondrial swelling; however, it is inhibited by rPKCε pretreatment of mitochondria and this inhibition is counteracted partly by tubulin, a VDAC inhibitor. (**A**) Rat liver mitochondria suspensions were preincubated for 25 min with rPKCε together with or without PMA, or rPKCα with PMA, and were subjected to BP treatment. Mitochondrial swelling was triggered with three times additions of 1 μM BP, as indicated, with 2 μM CsA when indicated. (**B**) Mitochondria were pretreated with or without rPKCε plus PMA in the presence or absence of 50 nM tubulin, and swelling was induced as in **A**. Data are representative of at least three separate experiments.

We examined the direct effect of PKCε on the PTP and performed a mitochondrial swelling assay with non-energized rat liver mitochondrial suspensions. Mitochondria were pretreated with recombinant PKCε (rPKCε) or recombinant PKCα (rPKCα) in a phosphorylation buffer containing ATP, Mg^2+^ and PMA for 25 min at 25 °C and were subjected to BP treatment. The results are shown in Fig. 7A. While control mitochondria underwent CsA-sensitive swelling in response to three additions of 1 μM BP, the mitochondria pretreated with PMA-activated rPKCε conferred resistance to BP-induced swelling to the comparable levels by CsA treatment; no such resistance was observed with mitochondria pretreated with inactive rPKCε (without PMA) or PMA-activated rPKCα. These findings suggest that the selective phosphorylation of mitochondrial protein(s) by PMA-activated rPKCε may desensitize the PTP to BP.

Because the VDAC is involved in BP-induced PTP opening and apoptosis, we suspected that the inhibition of swelling by rPKCε under a phosphorylation condition was mediated by the VDAC. If so, the presence of the VDAC inhibitor tubulin might eliminate the effect of rPKCε. To test this hypothesis, mitochondria were treated with PMA-activated rPKCε in the presence or absence of tubulin and then were subjected to BP-induced swelling. Figure 7B shows that rPKCε pretreatment with tubulin partially counteracted the rPKCε-induced suppression of BP-induced swelling, whereas tubulin alone had no effect on BP-induced swelling, suggesting that VDAC may involve the effect of rPKCε.

## Discussion

BP is cytoprotective at concentrations of < 1 μM under normal cell culture conditions (> 2% FBS). However, the present study showed that BP can induce cytochrome *c* mediated apoptosis at concentrations of 5−10 μM in low FBS conditions (< 0.1%) and that the apoptosis is regulated by CypD, and mediated by the prolonged induction of tPT (Fig. 1). The tPT may be mediated by the VDAC (Fig. 3) which is linked to the ANT (Fig. 4B) regulated by CypD activity (Fig. 1B). The switch of BP from a cytoprotective effect to an apoptotic effect may be primarily dependent on the absence of serum factors (Fig. 5), which results in the inactivation of PKC, especially PKCε, signaling to mitochondrial proteins (Fig. 6), including the VDAC (Fig. 7).

Because the sustained PT leads to mitochondrial energetic failure, it is generally thought to result in necrosis, but it also causes apoptosis^6,28,29,48^. It was previously proposed that apoptosis occurs when the PT affects a subset of mitochondria (since the ATP required for the execution of apoptosis is provided by the remaining intact mitochondria), while necrosis occurs when it affects most mitochondria (since the reverse activation of F-ATP synthase exhausts cellular ATP)^49^. In the present study, BP affected the whole mitochondrial population, but it caused apoptosis. This could be attributed to the BP-induced tPT retaining a low mitochondrial potential for a few hours (Fig. 1B). The low membrane potential prevents the reverse activation of F-ATP synthase and the exhaustion of cellular ATP, allowing the execution of apoptosis. The low membrane potential observed is in contrast with the high membrane potential during tPT in other experimental settings^6,7^. This could be due to a longer opening time or a higher opening frequency of the pore after BP treatment. The tPT triggered by BP may depend on CypD activity, as demonstrated by the inhibition of apoptosis by CsA and siRNA targeting CypD (Fig. 1A,B).

The low mitochondrial membrane potential associated with tPT may also be responsible for the extensive occurrence of mitophagy observed upon BP treatment (Fig. 2), as previously described^50^. Treatment with ALLN (a lysosomal protease inhibitor) delayed BP-induced apoptosis (Fig. 2B). Colchicine (a microtubule destabilizer) also delayed apoptosis (Fig. 2B), the formation of autophagolysosomes and cytochrome *c* release (Fig. 2Cii). These effects are probably due to the inhibition of microtubule-based mitochondrial trafficking. Colchicine increases free tubulin and prevents VDAC permeability in cells^51^ and Fig. 4Bi shows the tubulin inhibition of BP-induced PTP opening in isolated mitochondria. These data suggest that a potential increase in free tubulin upon colchicine treatment may interfere with BP-triggered tPT; however, this was not the case (Fig. 2Ci), probably because the free tubulin levels were insufficient for the inhibition of the action of BP.

Knockdown experiments with siRNA in HeLa cells indicated that VDAC1 and VDAC2 isoforms mediate BP-induced apoptosis (Fig. 3). This is consistent with the inhibition of BP-triggered mitochondrial swelling by tubulin (Fig. 7), whose specificity to the VDAC was demonstrated with the VDAC channel reconstituted into the planar lipid membrane^46,52^ and with cancer cells^51,53^. We did not evaluate VDAC3 in these experiments, because the import of NADH in the mitochondrial inter-membrane space is not possible when the porin 1 deficient yeast is complemented by VDAC3, but not by VDAC1 and VDAC2^54^. It has recently been reported that tubulin also blocks VDAC3^55^: the paper showed that even 85 nM of tubulin produced fewer blockage events in VDAC3 in comparison to those by 45 nM tubulin in VDAC1, indicating that VDAC3 is much less sensitive to tubulin than VDAC1. Using 50 nM tubulin we were able to fully inhibit BP-induced swelling, suggesting that there was no involvement of VDAC3. Furthermore, we observed no effect of transfection with VDAC1 siRNA, VDAC2 siRNA or both siRNAs on VDAC3 (Fig. 3A). Thus, it may be concluded that BP-induced tPT is mediated by the VDAC1 and VDAC2 isoforms.

Importantly, bongkrekate inhibited BP-induced pore opening in isolated liver mitochondria (Fig. 4B). This strongly suggests that ANT, and not ATP synthase, may form a pore for the BP-induced tPT, in line with two recent studies demonstrating the involvement of ANT in the PT^38,39^. Moreover, BP-induced mitochondrial swelling was observed to proceed at a very slow rate (Fig. 7) as compared with Ca^2+^-induced swelling (Fig. S1), and be of limited extent (Figs. 4Ai and 7), suggesting that the pore size may be smaller than that of the classic PTP channel, which is in good agreement with the properties of the CsA-sensitive ANT channel in ATP synthase c-subunit knockout mitochondria^38^.

BP-induced apoptosis was inhibited by 2% FBS, but not inhibited by 2% FBS in the co-presence of PKC inhibitors (Fig. 5A); it was also inhibited by PMA treatment (Fig. 5B). These data indicate the involvement of PKC signaling. PMA treatment did not block the tPT but prevented the complete loss of the mitochondrial membrane potential even at 3 h; the mitochondrial membrane potential remained high (19 ± 8%, representing the mean ± SD of the initial TMRM level) (Fig. 5C), compared with the complete loss at 3 h in control (Fig. 1Cii). This retention may be the primary mechanism for the apoptosis inhibition with PMA treatment, as it can block the shift to persistent PTP opening from tPT by preventing the loss of the mitochondrial matrix molecules essential for respiration via the opened pores^56^. The apoptosis inhibition by 2% FBS was accompanied by the inhibition of BP-induced tPT (unpublished data). This fact suggests that other signaling pathways, in addition to the PKC pathway, are needed for the complete inhibition of the tPT of BP. In PKC signaling, PKCε may be involved, as the overexpression of kinase-inactive PKCε counteracted the PMA-inhibition of BP-induced apoptosis by approximately half (Fig. 6). PKCε appears to directly phosphorylate mitochondrial proteins, since PMA-activated rPKCε (but not rPKCα) was able to inhibit BP-induced mitochondrial swelling to the same extent as CsA in isolated rat liver mitochondria (Fig. 7), which was attenuated by the VDAC inhibitor tubulin (Fig. 7B). These data imply a VDAC-mediated mechanism; however, whether PKCε directly phosphorylates VDAC or has an indirect effect on the VDAC through phosphorylating other mitochondrial proteins is unclear. It was reported that PKCε was able to phosphorylate VDAC directly and inhibit Ca^2+^-induced swelling of isolated cardiac mitochondria^33^, although these are challenged^57^. It should be noted that in contrast to the occurrence of Ca^2+^-dependent PTP opening in ischemia–reperfusion damage, the BP-induced tPT is apparently independent of the mitochondrial Ca^2+^ accumulation. In fact, Ca^2+^ preloading was not needed for the BP-induced tPT in isolated liver mitochondria (Figs. 4 and 7), and neither an increase in the cellular Ca^2+^ levels at 30 min after BP treatment nor any effect of BAPTA-preloading was observed in Hela cells (Supplementary Fig. S2). Furthermore, even though cellular Ca^2+^ might be increased by BP treatment, the rapid decrease in the mitochondrial membrane potential upon BP treatment is expected to prevent the mitochondria from taking up Ca^2+^.

In conclusion, our study demonstrated the pivotal role of tPT in BP-induced apoptosis, which is mediated by the ANT-associated pore, regulated by CypD. It is likely that whether BP-induced tPT is apoptotic or not depends on the PKC signaling, presumably, to the VDAC. These data will help define the cytoprotective mechanism by which BP protects against Ca^2+^-mediated oxidative cell death, which can be counteracted by CsA.

## Methods

### Animal studies

Wistar female rats were purchased from Japan SLC. Animal experiments were approved (#1800011) by the President of Josai International University after the review by Animal Care and Use Ethics Committee, and were carried out according to relevant guidelines and regulations.

### Apoptosis induction

HeLa cells that stably express GFP-tagged cytochrome *c* were cultured in basic medium (DMEM, 10 mM HEPES–NaOH, pH 7.4, 4 mM _L_-glutamine, penicillin and streptomycin), supplemented with 10% FBS, in 5% CO_2_ at 37 °C. Cells were seeded into 48-well plates (3 × 10^4^ cells/well) for cell death assay or 35-mm glass-bottomed dishes (3 × 10^5^ cells/dish) for confocal imaging. After overnight, cultures at ~ 50% cell confluency were washed three times with FBS-free basic medium, and changed to basic medium containing 0.1% FBS or 2% FBS for apoptosis induction by BP, which was synthesized (Chemical Soft, Japan). After 8 h cell death was assessed using a trypan blue exclusion test. When indicated, cells were pretreated with CsA (BIOMOL), FK506 (Calbiochem), ZVAD (Calbiochem), colchicine (Sigma), ALLN (Calbiochem), PMA (Sigma), BMI (Calbiochem) or staurosporine (Sigma) for 30 min before BP treatment. To downregulate cellular PKC, cells were treated for 24 h with 1 ng/ml PMA in basic medium containing 2% FBS. Apoptosis was induced by BP in the presence of 100 nM TMRM (Molecular Probes), and cells were subjected to confocal assessments of their membrane potential and cytochrome *c*-GFP. Mitophagy was assessed at 3 h after BP treatment in the presence of 100 nM LysoTracker green (Molecular Probes) and 100 nM MitoTracker red (Molecular Probes). To observe PTP opening, cells were incubated for 25 min at 37 °C with 0.25 μM calcein-AM (Molecular Probes), together with 1 mM CoCl_2_, 100 nM TMRM and 1 μM FK506 (MDR pump inhibitor) in HBSS buffer (Hank’s balanced salt solution containing 0.1% FBS and 25 mM HEPES–NaOH, pH 7.4), as described^6^. After washing apoptosis was induced with or without 5 μM CsA, 20 μM ZVAD, 0.3 μg/ml colchicine or 1 ng/ml PMA in HBSS buffer containing 1.5 mM CoCl_2_, 1 μM FK506 and 30 nM TMRM.

### Measurement of PTP opening in calcein-loaded mitochondria

Rat livers were homogenized in isolation buffer (0.3 M sucrose, 10 mM TES-KOH, pH 7.2) supplemented with 1 mM EGTA-KOH. After centrifugation at 1,000 × *g* for 5 min at 4 °C, supernatants were centrifuged at 10,000 × *g* for 5 min. The pellet resuspended in 0.5 ml isolation buffer was layered on a gradient of 0.2 ml 18%, 0.5 ml 40%, and 0.3 ml 60% Percoll (top to bottom). Following centrifugation at 10,000 × *g* for 5 min, the mitochondrial fraction was recovered from 40 to 60% Percoll interphase, washed, and stored at a protein concentration of 13 mg/ml (Biuret assays). For calcein loading, mitochondria (1 mg/ml) were treated with 0.25 μM calcein-AM on ice for 15 min, washed twice by centrifugation at 7,000 × *g* for 3 min and stored at 13 mg protein /ml.

For mitochondrial suspension experiments, an aliquot (10 μl) of calcein-loaded mitochondria was added into 1 ml of assay buffer (0.25 M sucrose, 20 mM Tris-MOPS, pH 7.4, 20 μM EGTA, 0.1 mM KP_i_ and 3 mM MgCl_2_, 5 mM succinate and 50 nM TMRM) (Fig. 4A).

Agar-embedded mitochondria were prepared^45^: aliquots (5 μl) of mitochondrial suspensions, which were prepared by diluting calcein-loaded mitochondria 4 times in assay buffer containing DMSO, 3 μM CsA, 50 μM bongkrekate (Calbiochem) or 50 nM tubulin (Cytoskeleton), were mixed with 5 μl of 2% (w/v) type VII agarose in assay buffer (37 °C), with half of these mixtures placed in circular areas with a diameter of 15 mm circumscribed with a PAP pen on a 24 mm × 32 mm cover glass. After solidification, agar was covered with 0.3 ml of assay buffer. Tubulin was prepared according to the manufacturer’s instruction.

### RNA interference with siRNA

CypD-targeting siRNA was purchased from Ambion. The siRNA targeting sequences^58^ were: nonsense (5′-CGUACGCGGAAUACUUCGA-3′), CypD (5′-GGACUCUAAUACCUGUUUAtt-3′). VDAC1- and VDAC2-targeting siRNAs were purchased from Qiagen. The siRNA target sequences^59^ were: nonsense (5′-AAUUCUCCGAACGUGUCACGU-3′), VDAC1, (5′-ACACUAGGCACCGAGAUUAUUAtt-3′) and VDAC2 (5′-AAUACAAGUGGUGUGAGUAtt-3′). The nucleotide sequences of the three VDAC isoforms show high homology. However, the siRNA targeting VDAC1 bears 7 and 5 nucleotide mismatches, respectively, in comparison to its corresponding region of VDAC2 (5′-CTCTGGGAACAGAAATCG-3′, the different nucleotides are underlined) and to that of VDAC3 (5′-ACTCTAGGGACAGAAATCT-3′). The siRNA for VDAC2 bears 5 and 6 mismatches, respectively, in comparison to that of VDAC1 (5′-AGTACAGATGGACTGAGTA-3′) and to that of VDAC3 (5′-AATATAAGGTCTGTAACTA-3′). Thus the off-target effects on non-target VDAC isoforms seems unlikely based on the siRNA sequences, which were confirmed by immunoblotting experiments (Fig. 3A). HeLa cells were cultured for 7−8 h until 20%-30% confluency in 48-well plates in basic medium supplemented with 10% FBS. After the medium was replaced with basic medium containing 0.5% FBS, transfection of cells with 20 nM siCypD, 5 nM siVDAC1 + 5 nM control siRNA, 5 nM siVDAC2 + 5 nM control siRNA, 5 nM siVDAC1 + 5 nM siVDAC2, and 10 nM control siRNA was performed using jetPRIME (Polyplus-transfection SA). After 36 h, apoptosis was then induced by BP.

### A mutant PKCε gene transfection

HeLa cells (6 × 10^4^ cells/35-mm dish) were infected for 24 h at a multiplicity of infection of 100 plaque-forming units per cell with control adenovirus vector or adenovirus vector encoding a kinase-defective mutant PKCε in which Lys^437^ was replaced by methionine^47^. After seeded in 96-well plates (6 × 10^3^ cells/well), cells were cultured overnight in basic medium containing 10% FBS, which were subsequently subjected to BP-induced apoptosis.

### Immunoblotting

Commercially available primary antibodies were used: rabbit polyclonal antibodies against CypD (239784, Calbiochem), PKCε (sc-214, Santa Cruz), actin (sc-1615, Santa Cruz), VDAC2 (ab47104, Abcam) and VDAC3 (ab130561, Abcam), and a mouse monoclonal antibody against VDAC1 (ab14734, Abcam). Cells were washed 3 times in PBS in 48-well plates and each well on a heat block was treated with 10 μl of boiled sample buffer (2% SDS, 1% NP40, 5% sucrose in 62 mM Tris–HCl, pH 6.8) containing protease inhibitors (50 μM PMSF, 50 μM leupeptin, 1 μg/ml aprotinin, and 0.5 μM pepstatin). After scraping, the recovered cells were further disrupted by sonication at output 10 for 30 s with a TOMY ultrasonic disruptor UD-200 and centrifuged at 20,000 × *g* for 3 min at 4 °C. The supernatants, supplemented with 2-mercaptoethanol and bromophenol blue, were again boiled and were loaded into a 12% (for CypD), 10% (for VDAC) or 8% (for PKCε) sodium dodecyl sulfate–polyacrylamide gel electrophoresis. The proteins were transferred to a PVDF membrane. Membranes were blocked with 5% non-fat milk for 2 h in PBS, and then incubated for 1.5−2 days at room temperature with specific primary antibodies: After washing with 0.1% tween 20 in PBS, blots were incubated overnight with biotinylated secondary antibodies and detected using the ABC method (PK-6100, Vector Labs).

### Mitochondrial swelling

Mitochondrial suspensions (0.8 mg/ml) were incubated with or without 100 nM tubulin at 25 °C in a buffer consisting of 10 mM Tris–HCl (pH 7.4), 120 mM KCl, 20 mM MOPS, and 0.5 mM KH_2_PO_4_. After 5 min, 25 μl aliquots of the suspensions were mixed at 25 °C with 25 μl aliquots of phosphorylation cocktail (0.4 mM ATP, 20 mM MgCl_2_ and 2 μM PMA) with or without 4 μg/ml rPKCε (Invitrogen) or 4 μg/ml rPKCα (Calbiochem). After 25 min, the mixtures (50 μl) were diluted by adding 100 μl of swelling buffer (0.3 M sucrose, 10 mM TES-KOH, pH 7.2, 10 mM KCl, 1 mM MgCl_2_, 25 μM EGTA and 0.2 mM KP_i_), and centrifuged at 10,000 × *g* for 5 min. The pellets were resuspended in 50 μl of swelling buffer, and then halved mixtures were added to cuvettes containing 1 ml of swelling buffer supplemented with 0.1 μM FCCP (Sigma) (for complete dissipation of the membrane potential). Swelling was induced by adding 1 μM BP three times at 2, 3 and 4 min and was detected spectrophotometrically at 520 nm (FP-6200, JASCO International).

### Confocal microscopy

Fluorescent signals were monitored using Olympus FV-300 and FV-1000 laser scanning confocal microscopes equipped with a × 40 and × 60 oil immersion objective for 35-mm glass-bottomed dishes and cover glasses, respectively. GFP, LTG and calcein were then excited by 488 nm and fluorescence was detected with 505−530 nm band-pass filter; TMRM and MTR were excited at 543 nm and fluorescence was detected with a 560−620 nm filter. For the acquisition and quantification of confocal signals, calcein, cytochrome *c*-GFP, and TMRM fluorescence signals were calculated by subtracting the background signals from signals in regions of interest, manually applied using the Olympus FV10-ASW software program. The background signals for TMRM were obtained after treatment with 0.5 μM FCCP.

### Statistics

All results are representative of at least three different mitochondrial isolations and at least three cell death experiments. Data were statistically analyzed using a 1-way or 2-way ANOVA, followed by a multiple comparison analysis with a *t*-test with Bonferroni’s correction.

## Supplementary information

Supplementary information 1.

Supplementary information 2.

Supplementary file3
