# Supplementary material for: Bisindolylpyrrole triggers transient mitochondrial permeability transitions to cause apoptosis in a VDAC1/2 and cyclophilin D-dependent manner via the ANT-associated pore
Source: Sci Rep. 2020 Oct 12;10:16751. doi: 10.1038/s41598-020-73667-z (PMC7552391; doi:10.1038/s41598-020-73667-z)
Supplement: Supplementary file 1 — Supplementary information 1. [file 41598_2020_73667_MOESM1_ESM.doc]

**Bisindolylpyrrole triggers transient mitochondrial permeability transitions to cause apoptosis in a VDAC1/2 and cyclophilin D-dependent manner via the ANT-associated pore.**

Masami Koushi^1^, Yasunori Aoyama^1^, Yoshiko Kamei^1^, and Rei Asakai^1^*

^1^Department of Morphophysiology, Faculty of Pharmaceutical Sciences, Josai International University, 1 Gumyo, Togane, Chiba 283−8555, Japan

*Correspondence should be addressed to Rei Asakai, Department of Morphophysiology, Faculty of Pharmaceutical Sciences, Josai International University, 1 Gumyo, Togane, Chiba, 283−8555, Japan.

Tel: +81−475−53−4588; Fax: +81−475−53−4581

E-mail: [asakai@jiu.ac.jp](mailto:asakai@jiu.ac.jp)

# Supplementary Information

**Materials and Methods**

*Cellular Ca^2+^ monitoring with Fluo-8*

# HeLa cells were incubated for 30 min with 4 μM Fluo-8-AM (AAT Bioquest) with or without 50 µM BAPTA (1,2-bis(*o*-aminophenoxy)ethane-N,N,N’,N’-tetraacetic acid)-AM in HBSS containing 0.1% FBS. After rinsing with DMEM containing 0.1% FBS, and were subjected to treatment with 5 μM A23187 (Calbiochem) as a positive control for calcium increase. Cells were also treated with BP.

*Swelling in BAPTA-preloaded liver mitochondria*

# To load BAPTA, liver mitochondrial suspensions (10 mg wet weight/ml) in stock buffer containing 50 μM EGTA were incubated for 30 min at 25 °C with 100 µM BAPTA-AM. After centrifugation at 5,000 × *g* for 3 min, pellets were resuspended in stock buffer (100 mg wet weight /ml). The suspension (10 µl) was then added to 1 ml of swelling buffer supplemented with 0.1 μM FCCP in a non-energized condition. Swelling was initiated by adding 5 μM ETH129 (Sigma) and 10 μM CaCl_2_ or BP three times (3, 2, and 5 μM at 0. 5, 2.5, and 4.5 min respectively).

**Legends**

# Figure S1. Ca^2+^ (200 μM)-induced PTP opening in agar-embedded rat liver mitochondria energized with succinate, preloaded by TMRM and calcein. Representative images for TMRM and calcein signals of mitochondria in the same areas; note that both signals dropped within 3 min. Scale bar: 5 μm. Images were digitized using FV10-ASW software version 4.2a (https//www. olympus-lifescience.com).

# Figure S2. (A) Effect of BP on HeLa cells preloaded with Fluo-8. While 1 µM A23187 treatment increased the Ca^2+^ signals within 30 min, as indicated by an increase in Fluo-8 fluorescent signals, which was eliminated by the presence of BAPTA (i), treatment with 7.5 µM BP had no effect at 3 h (ii). (B) No effect of BAPTA preloading on BP-initiated liver mitochondrial swelling, while BAPTA preloading inhibited the swelling induced by ETH129 plus CaCl_2_. Scale bar: 10 μm. Images were digitized using FV10-ASW software version 4.2a (https//www. olympus-lifescience.com).
