# Supplementary figures and images for: Bisindolylpyrrole triggers transient mitochondrial permeability transitions to cause apoptosis in a VDAC1/2 and cyclophilin D-dependent manner via the ANT-associated pore
Source: Sci Rep. 2020 Oct 12;10:16751. doi: 10.1038/s41598-020-73667-z (PMC7552391; doi:10.1038/s41598-020-73667-z)

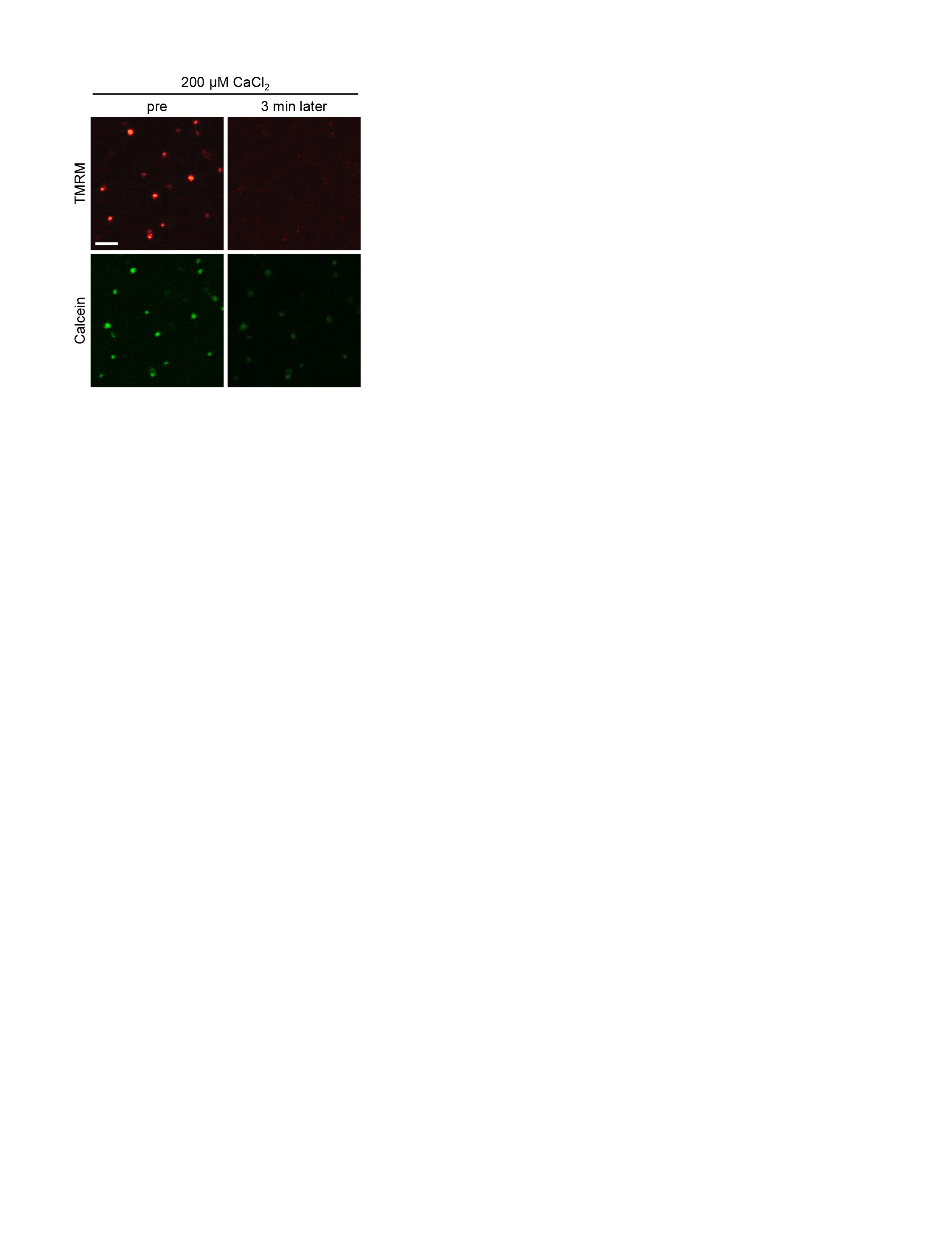

Supplement: Supplementary file 2 — Supplementary information 2. [file 41598_2020_73667_MOESM2_ESM.jpg]

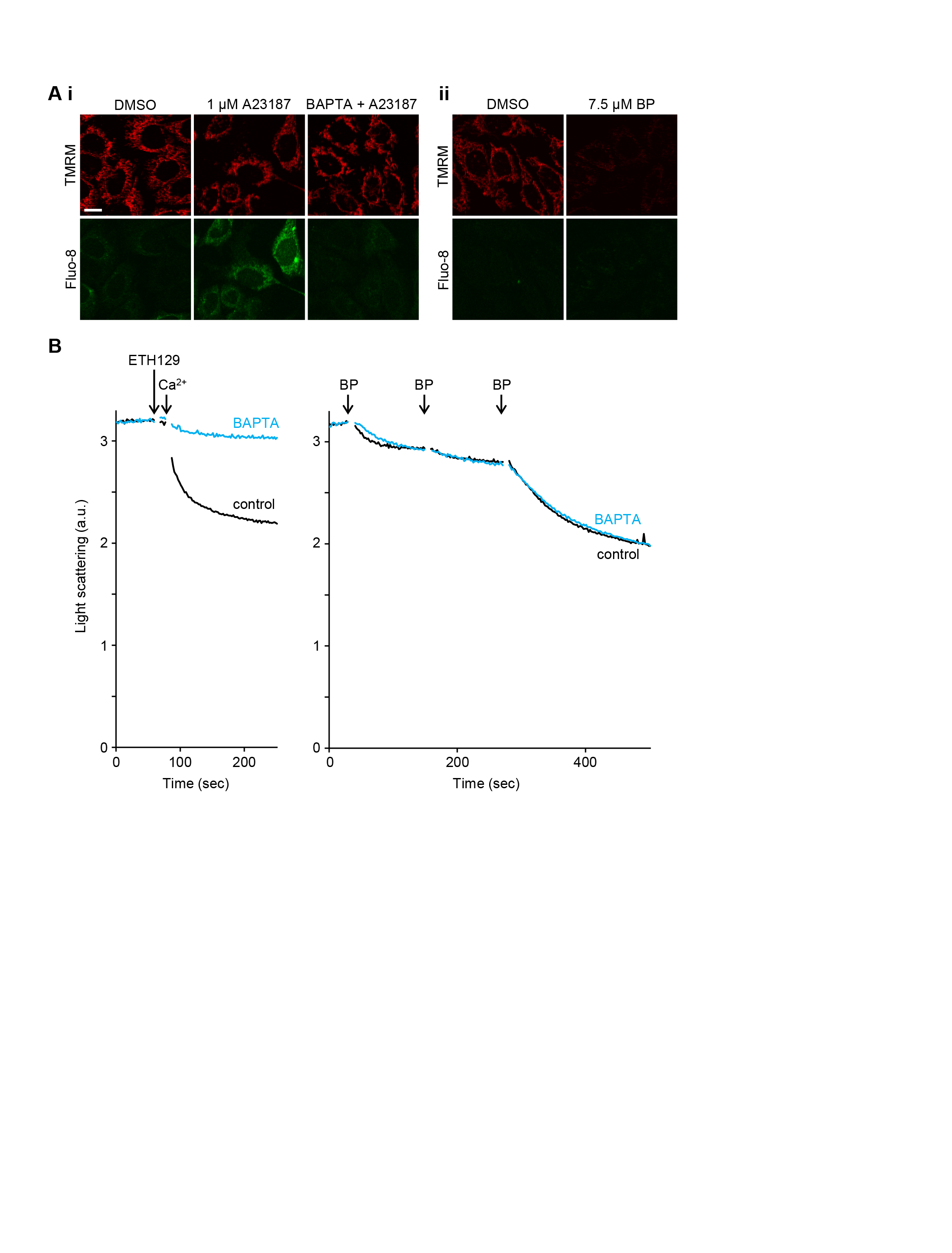

Supplement: Supplementary file 3 — Supplementary file3 [file 41598_2020_73667_MOESM3_ESM.jpg]
